# A dissociation between engagement and learning: Enthusiastic instructions fail to reliably improve performance on a memory task

**DOI:** 10.1371/journal.pone.0181775

**Published:** 2017-07-21

**Authors:** Benjamin A. Motz, Joshua R. de Leeuw, Paulo F. Carvalho, Kaley L. Liang, Robert L. Goldstone

**Affiliations:** 1 Department of Psychological and Brain Sciences, Indiana University, Bloomington, Indiana, United States of America; 2 Cognitive Science Program, Indiana University, Bloomington, Indiana, United States of America; 3 Cognitive Science Department, Vassar College, Poughkeepsie, New York, United States of America; 4 Human-Computer Interaction Institute, Carnegie Mellon University, Pittsburgh, Pennsylvania, United States of America; Waseda University, JAPAN

## Abstract

Despite widespread assertions that enthusiasm is an important quality of effective teaching, empirical research on the effect of enthusiasm on learning and memory is mixed and largely inconclusive. To help resolve these inconsistencies, we conducted a carefully-controlled laboratory experiment, investigating whether enthusiastic instructions for a memory task would improve recall accuracy. Scripted videos, either enthusiastic or neutral, were used to manipulate the delivery of task instructions. We also manipulated the sequence of learning items, replicating the spacing effect, a known cognitive technique for memory improvement. Although spaced study reliably improved test performance, we found no reliable effect of enthusiasm on memory performance across two experiments. We did, however, find that enthusiastic instructions caused participants to respond to more item prompts, leaving fewer test questions blank, an outcome typically associated with increased task motivation. We find no support for the popular claim that enthusiastic instruction will improve learning, although it may still improve engagement. This dissociation between motivation and learning is discussed, as well as its implications for education and future research on student learning.

## Introduction

Enthusiasm is widely believed to be fundamental to effective teaching. Expert teachers routinely promote the crucial importance of enthusiastic instruction for inspiring student interest, motivation, and attention toward the course topics and activities (e.g.,[1–3]). In his early survey of teaching behaviors, Barr [4] observed that enthusiastic “energy and vitality” were the principal differentiating characteristics of “good” teachers. In McKeachie’s popular guidebook of teaching tips, the lecturer’s primary role in higher education is reduced simply: “to communicate the teacher’s enthusiasm for the subject.” ([5], p. 71). Undergraduate students also rank enthusiasm among the most important and valuable properties of instructors [6, 7], and accordingly, student ratings of teachers’ enthusiasm represent a dominant factor in end-of-semester evaluations [8, 9]. Moreover, instructors who receive uniformly high marks on student evaluations also tend to rank particularly high on extraversion personality scales, marked by behavioral traits such being excited, outgoing, and playful [10, 11].

One might assume that the importance of enthusiasm, as assessed by expert teachers and students alike, would be supported by strong evidence of its relationship to student learning. However, in contrast to these strong intuitions, research results are mixed. Even while enthusiastic teaching reliably correlates with student interest, involvement, and enjoyment (reviewed in [12]; c.f. [13]), a clear connection between enthusiasm and student achievement (test performance, course grades, etc.) has not been identified.

Mastin [14] conducted the earliest cited experimental manipulation of teacher enthusiasm. A teacher gave two lecture-style lessons to 6th and 7th graders, one-week apart, regarding two different ancient civilizations. One lesson was delivered in an indifferent manner, and the other in an enthusiastic manner, counterbalanced for order of presentation and the civilization described. On a subsequent memory test, 19 of the 20 classrooms had better performance scores for the civilization that had been presented enthusiastically. Additional published experiments have similarly observed improved learning outcomes under enthusiastic teaching conditions [15–19], however, some published studies have also failed to observe these effects [20–23], and some suggest that any effect of enthusiasm may become negligible when students are given an extrinsic incentive to learn the material [24, 25]. Interpretation of these mixed results is difficult because many of these studies lacked random assignment of learners to classroom experimental conditions [15, 17, 20–24], or because additional factors (such as the amount of instructor-student interaction) were comingled in the enthusiasm condition [19, 26]. Unfortunately, experimental attempts that included careful random assignment of students to controlled enthusiasm conditions have also observed no significant effects, or very limited effects, of enthusiasm on learning [27–29].

These mixed results have been observed under a range of operationalizations of enthusiasm, from a specific set of nonverbal gestures [30], to broader conceptualizations including the expression of humor, energy, and charisma [31], such that inconsistent findings cannot be conveniently attributed to semantic differences in how different researchers define or manipulate enthusiasm. Effects on student achievement, or lack thereof, are not limited to some narrow set of instructor actions or behaviors. This should make sense because different expressions of enthusiasm are not independent from each other. Indeed, factor analyses have observed that expressive qualities tend to covary in authentic instruction [9, 32], and for this reason, researchers have suggested that inclusive and multifaceted definitions of enthusiasm are preferable [12, 33].

Theoretically, the effect of enthusiastic instruction should be to improve a student’s motivation to do something [34]. But past experimental research on enthusiasm has emphasized the teacher’s enthusiastic delivery of *information* to be learned, and not the enthusiastic delivery of *instructions* for a learning activity. Considering that self-regulated learning is playing an increasingly vital role in educational systems, enthusiastic instruction should motivate a student’s independent studying behaviors, not merely animate the to-be-learned material. That is, for situations in which students will be assigned to seek out their own individualized learning materials, instructors will often be in the position of being able to vary their enthusiasm in exhorting the student to go forth and learn, but will not be able to directly affect the enthusiasm exhibited in the materials themselves. In support of this perspective, there have been some indications that a teacher’s enthusiasm will only affect memory performance when students consider themselves to be in control of their own learning [35, 36].

The rather inconclusive state of research on instructor enthusiasm stands in stark contrast to recent developments in cognitive learning interventions. Memory researchers have amassed a considerable catalogue of studying techniques that improve learning, and generalize to a range of educational applications [37]. For example, the *spacing effect* (memory improvement when repetition of study items is distributed over time rather than massed; [38]), one of the largest and most reliable effects in the learning and memory literature [39], has been repeatedly demonstrated to improve learning and student achievement in authentic educational contexts (e.g., [40–45]). But these cognitive techniques, despite their reliability and effectiveness, remain relatively unknown to educators [46], and students often do not recognize their practical value [47, 48]. Enthusiasm, on the other hand, may be a widespread pedagogical stratagem in education, but its relationship to student learning lacks for unequivocal empirical support.

The lack of consistent findings may be, in part, because past researchers have aimed to quantify enthusiasm’s effect in naturalistic educational settings. While these efforts toward ecological validity are laudable [49, 50], the current study instead aims to quantify the effect of instructor enthusiasm in a delayed-recall laboratory memory experiment. Advantages of this approach include the ability to randomly-assign individual participants to different instructional conditions (thus avoiding sampling bias of classroom groups, as well as the potential for learner-to-learner interactions that are not of interest to the current study), to tightly control the stimuli and learning materials (thus avoiding confounds from teacher-student interactions that might be concomitant with face-to-face enthusiastic teaching), and to emulate the individualized and self-paced instructions that are characteristic of contemporary blended learning environments. And in doing so, the effect of enthusiasm can also be measured alongside the effect of a cognitive manipulation, such as the spacing effect.

Considering that enthusiasm occupies a high pedestal among teaching behaviors, and that the research foundation for its status is not particularly sturdy, the literature on its relationship to student achievement can clearly benefit from a controlled memory experiment. The primary goal of the current study is to address this need, examining whether enthusiastically-delivered instructions can affect performance in a laboratory memory task. Because enthusiasm has been claimed to affect both learning and motivation [51], we focus on two distinct measures of task performance: recall accuracy (the number of study items correctly remembered after a delay) and recall quantity (the total number of responses, correct and incorrect, provided at test; which has been observed to be sensitive to experimental manipulations of motivation and encouragement [52, 53]). We also seek to quantify these effects of instructor enthusiasm alongside the effect of temporally spacing the repetition of study items (the spacing effect), a known cognitive technique for memory improvement. By manipulating instructional enthusiasm in two different sequence conditions, we add greater contextualization to our assessment of the main effect of enthusiasm, given different study tactics. Moreover, this direct contrast between instructional behaviors and study sequencing may help provide a better understanding of the relative efficacy of these two educational strategies for improving learning and memory.

## Experiment 1 (Exploratory study)

Experiment 1 was intended to provide a preliminary examination of whether enthusiastically-delivered instructions would affect performance on a typical paired-associates delayed-recall memory task. In order to maximize experimental control of the instructor’s delivery, we made two video recordings of the task instructions presented by the same instructor, one was enthusiastic, the other neutral. Participants watched either video just prior to studying the paired associates, one pair at a time, in a computer-guided sequence that either distributed the study items uniformly (spaced) or repeated the study items back-to-back (massed). One week later, participants returned to the lab for a recall test. All materials, including videos, raw data, and analysis scripts, are available at http://osf.io/j2h8y/.

### Method

#### Ethics statement

The experimental procedures, materials, and recruitment protocols were approved by the Indiana University Institutional Review Board. All participants provided informed consent electronically at the start of the experiment (see Procedure section below).

#### Participants

Eighty-six undergraduate students at Indiana University Bloomington volunteered to participate in this two-session study toward completion of their Introductory Psychology experiment participation requirements. Sample size for this exploratory study was not predetermined, and data collection was stopped at the end of the academic term. Participants were informed in the experiment instructions that, in addition to the credits toward their course requirement, they would also be paid a $0.25 bonus for each word pair correctly recalled (the maximum bonus was $9) at the end of the second session. Seven participants were excluded from analyses because they did not return to complete the second session (N = 5), there was an error during data collection (N = 1), or because the participant failed to complete the study phase in the allotted time (N = 1); leaving 79 participants in total, 48 females (by self-report; 61%), between the ages of 18 and 24 years (*M* = 19.4, *SD* = 1.2). Sixty-one of these participants reported being native English speakers (77%), and the remaining non-native English speaking participants had been speaking English for an average of 12.3 years (*SD* = 3.4). No Indonesian speakers volunteered to participate in this study.

#### Stimuli

We selected 36 English-Indonesian word pairs from a larger set of 96 word pairs used in [54]. Based on the norming data provided, the selected words had recall accuracy ranging from 43% to 100%.

#### Video recorded instructions

We wrote comprehensive task instructions for this memory experiment in two different scripts; one was written so as to demonstrate enthusiasm for the participant’s task performance, and the other was neutral. Each of these scripts contained the same structure and basic information, with an instructor initially welcoming the participant to the study, providing background information, describing step-by-step procedures, and so on. The enthusiastic script was written to express charisma, personality, and humor (Williams and Ware, 1976), and accordingly, it included language that was more active, personally relevant, encouraging, and specific. The neutral script contained all the same task directions, but it was written in a more general and impersonal tone than the enthusiastic script, including passive phrases and unadorned straightforward statements and instructions.

We posted a job advertisement to graduate students enrolled in Indiana University’s Masters of Fine Arts (MFA) acting program, to recruit a paid performer for these scripts. We ultimately hired a talented female actor, Ashley, who had experience making professional training videos, and also had extensive experience teaching undergraduates. She was asked to deliver the enthusiastic script with varied vocal inflexion, demonstrative gesturing and movements, and emotive facial expressions [30], imagining an enthusiastic and dynamic teacher who is well-liked by her students. She prepared the neutral script without these expressive qualities, imagining a teacher who is polite and speaks clearly, but who isn’t expressive or particularly invested in inspiring her students. We described the experiment to the actor, and she confirmed that she would be able to comfortably assume these instructional roles in a realistic manner. She memorized the scripts, and we staged and recorded the instructions in the same testing room that participants would be performing the experiment.

**Fig 1:**
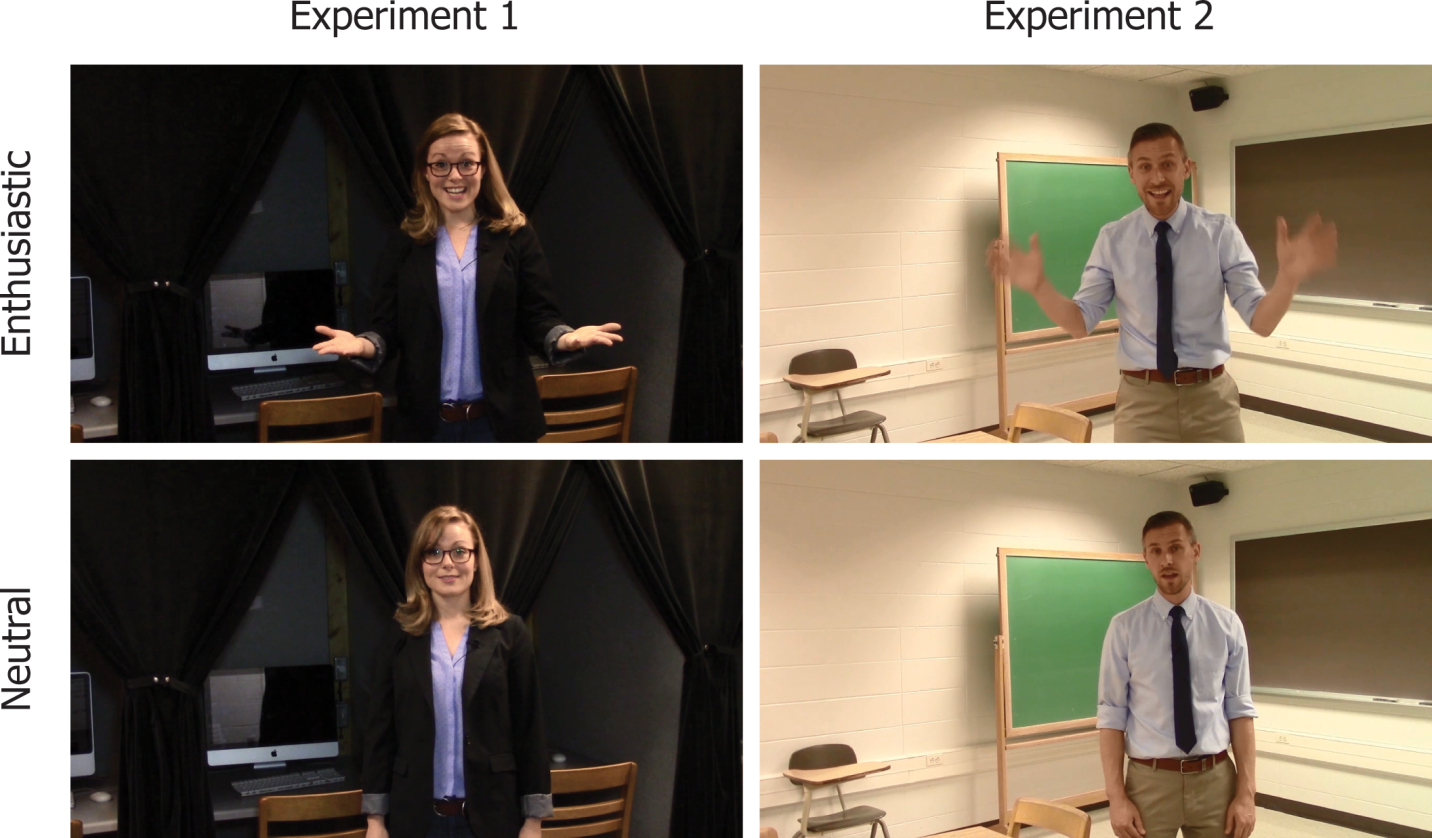
Examples from instructional videos. Representative stills at equivalent moments in enthusiastic and neutral instructional videos. Experiment 1 stills are from the video shown halfway through the study session. Experiment 2 stills are when providing background information about Indonesian at the start of the study session. Full videos available at http://osf.io/j2h8y/.

The total duration of all recorded video segments in the enthusiastic condition was 4 minutes 37 sec, and 4 minutes 6 seconds in the neutral condition (see Fig 1 for screenshots; the individuals shown in this figure have given written consent to publish these images).

#### Procedure

When initially signing-up for the experiment, participants scheduled two separate appointments (first for a study session, and then for a recall test session), with the constraint that the second session take place 5 to 9 days after the first session.

When they arrived for the first session, participants were greeted by an undergraduate research assistant, who led them to individual testing cubicles, and initialized the experiment on a desktop computer with comfortable headphones. Upon initializing, the experiment script (written with jsPsych [55]) automatically assigned each participant to an enthusiasm condition (enthusiastic or neutral) and a sequence condition (massed or spaced), such that condition assignments were unknown to the research assistant. Participants clicked to provide informed consent on the first screen, and then the study began with a video of the instructor introducing herself, welcoming them to the experiment and explaining the task, either in an enthusiastic or a neutral manner. Although the delivery of the instructions differed between these two conditions, both introductory video clips informed participants that they would study pairs of Indonesian and English words, that their goal was to learn the translation of each Indonesian word, and that they would receive a monetary bonus of $0.25 for each Indonesian word they correctly translated during the second session. Participants could not control video playback, all videos were presented exactly once, each in its entirety.

Following these initial instructions, participants then studied a total of 144 virtual flashcards (36 word pairs, ordered randomly, repeated 4 times each), each with an Indonesian word on the front and the corresponding English translation on the back. Participants clicked the flashcard to flip it and reveal the English translation. Participants were only able to flip each card once, but were free to spend as much time as they wished on each side of the flashcard (with the constraint that the entire study phase could not last more than one hour). Participants in the massed condition saw the 4 repetitions of each word pair back-to-back, whereas the other half of participants (spaced condition) saw each repetition only after studying all other word pairs.

A short break was included after card 72 (halfway through the 144 trials). During the break, participants watched a brief video informing them that it was the halfway point and reminding them to take their time and stay focused. This video was either enthusiastic or neutral, matching the participant’s initial condition assignment.

After participants had studied all flashcards, another video (either enthusiastic or neutral) asked them to complete a short demographic survey, and a final video (again, either enthusiastic or neutral) thanked them and asked them to confirm their next appointment with the research assistant. These extra procedural steps were intended to reduce the likelihood that participants would write down any word pairs immediately following the study phase.

Upon returning to the lab a week later, an undergraduate research assistant directed each participant to a computer where the recall task would take place; whenever possible we used the same cubicle that the participant used in the study session. A short video (either enthusiastic or neutral, again according to the participants’ initial condition assignments) welcomed them back to the experiment and described the recall task. All participants saw the 36 Indonesian words in random order, one at a time, in the center of the screen and had to type the English translations. The recall task was self-paced and participants were allowed to leave the field empty if they did not remember the translation, however, participants could not go back to change previously-submitted responses. Slight misspellings and near-synonyms were tolerated for participant payment bonuses, but only exact matches with the previously-studied English translation were counted as correct for data analysis. After the recall task, participants completed a short questionnaire, overall accuracy was then displayed on the screen, and the performance bonus was calculated and processed. This final questionnaire included three items adapted from the enthusiasm scale of a standardized student evaluation instrument (SEEQ; [56]), “Ashley was enthusiastic about this activity,” “Ashley was dynamic and energetic in conducting this activity,” and “Ashley’s style of presentation held my interest” (rated on a 5-point scale of agreement). We also included a question about the memorability of the instructor (“I have strong memories of the video of Ashley”), and participants also self-assessed their own task motivation (“I felt motivated to do well in this experiment”).

#### Bayesian data analysis

We used Bayesian estimation methods for all our data analyses, which has different analytical goals than null hypothesis significance testing [57]. Rather than controlling long-run false-positive rates (as in null hypothesis significance testing), this analysis produces an informative posterior distribution of the most credible descriptions of the data. A major advantage of this approach is that the analysis directly describes an estimate of uncertainty, while avoiding conceptual problems with frequentist alternatives like confidence intervals [58, 59]. Bayesian estimation involves specifying an analytical model of how experimental effects influence an outcome measure, then estimating credible parameter values for the model given the observed data, and ultimately investigating the distribution of credible parameter values to make inferences about experimental effects (for an accessible introduction to Bayesian estimation, see [60]).

Our analysis model for test performance assumes that the number of correct responses, *y*, for a given participant, *p*, is generated by a binomial distribution with probability of success, *θ*_*p*_, out of 36 English-Indonesian test item trials.

$$y_{p}∼Binomial(\theta_{p},\;36)$$

We assume that *θ* values for each participant are drawn from a beta distribution that is specific to participants who had the same kind of instruction (enthusiastic or neutral, *e*) and sequence of study items (massed or spaced, *s*). The beta distribution’s shape parameters, *α* and *β*, were parameterized in terms of the mode (*μ*) and concentration (*κ*) of the distribution [59].

$$\theta_{p}∼Beta(\alpha_{e,s},\beta_{e,s})$$

$$\alpha_{e,s}=\mu_{e,s}\times (\kappa_{e,s}-2)+1$$

$$\beta_{e,s}={(1-\mu }_{e,s})\times (\kappa_{e,s}-2)+1$$

The parameters *μ* and *κ* (the mode and concentration of item response, respectively) are our primary interest in describing the group-level effects of enthusiasm and study sequence. The parameter *μ* is a direct estimate of overall test performance (out of 36 trials) in an enthusiasm/sequence condition (denoted by the subscripts *e* and *s*), and *κ* is an estimate of the group-level variance of test performance for that condition. We put vague priors on *μ* (as drawn from a uniform beta distribution) and *κ* (as drawn from a gamma distribution), informed only by the scale of the data.

$$\mu_{e,s}∼Beta(1,1)$$

$$\kappa_{e,s}∼Gamma(mode=10,\;sd=100)$$

For the analysis of questionnaire responses (participant’s subjective ratings of the instructor’s enthusiasm and of their own motivation), we treated rating scales as numeric measures, and used a standard Bayesian hierarchical model for robust comparison of two groups [61]. In this model, the mean of both groups combined is used as the prior for each individual group mean, and the prior for each group’s standard deviation is 5 times the full sample’s standard deviation.

We estimated the posterior distributions of the models using JAGS [62] and the runjags package [63] for R [64]. We used four parallel Markov chain Monte Carlo (MCMC) runs; each of the four chains had 1,000 steps of adaptation and 4,000 steps of burn-in before sampling 60,000 times from the posterior distribution. The final sample was thinned to every three steps, for a total sample of 80,000 (4 chains x 20,000 samples). The effective sample size for the parameters of interest were no less than 20,000, well above the 10,000 recommended by Kruschke [59]. The Gelman-Rubin convergence diagnostic [65] was 1.0 for all parameters, indicating proper convergence of the MCMC chains.

The posterior is a probability distribution of credible parameter estimates for the model, given the observed data and the priors. A posterior distribution’s 95% highest density interval (HDI) defines the boundaries of the most credible estimates of the parameter, so that the values inside the HDI are more probable than those outside. When a parameter’s 95% HDI overlaps zero, it remains reasonably probable that the parameter is zero, or that the estimate is so close to zero that it is not theoretically meaningful.

### Results

Participants in the enthusiasm condition rated the simulated instructor, Ashley, as marginally more enthusiastic (*M* = 3.4. *SD* = 0.5; on a zero-to-4-point scale, the mean of the 3 SEEQ items) than participants in the neutral condition (*M* = 3.2, *SD* = 0.6), but the 95% HDI of the posterior distribution of this difference of means included zero and values close to zero (mode = 0.203; 95% HDI: -0.0474 to 0.501). On another item, “I have strong memories of Ashley,” participants in the enthusiasm condition also provided marginally-higher ratings (*M* = 3.0, *SD* = 0.8; on a 1-to-5-point scale) than the neutral condition (*M* = 2.6, *SD* = 1.0), but again, the posterior distribution of the difference of these means overlapped zero (mode = 0.346; 95% HDI: -0.0747 to 0.779). There was no difference between enthusiasm conditions in participants’ responses to the item “I felt motivated to do well in this experiment” (both enthusiastic and neutral were *M* = 2.8, *SD* = 0.82 in enthusiasm condition, *SD* = 0.72 in neutral condition).

**Fig 2:**
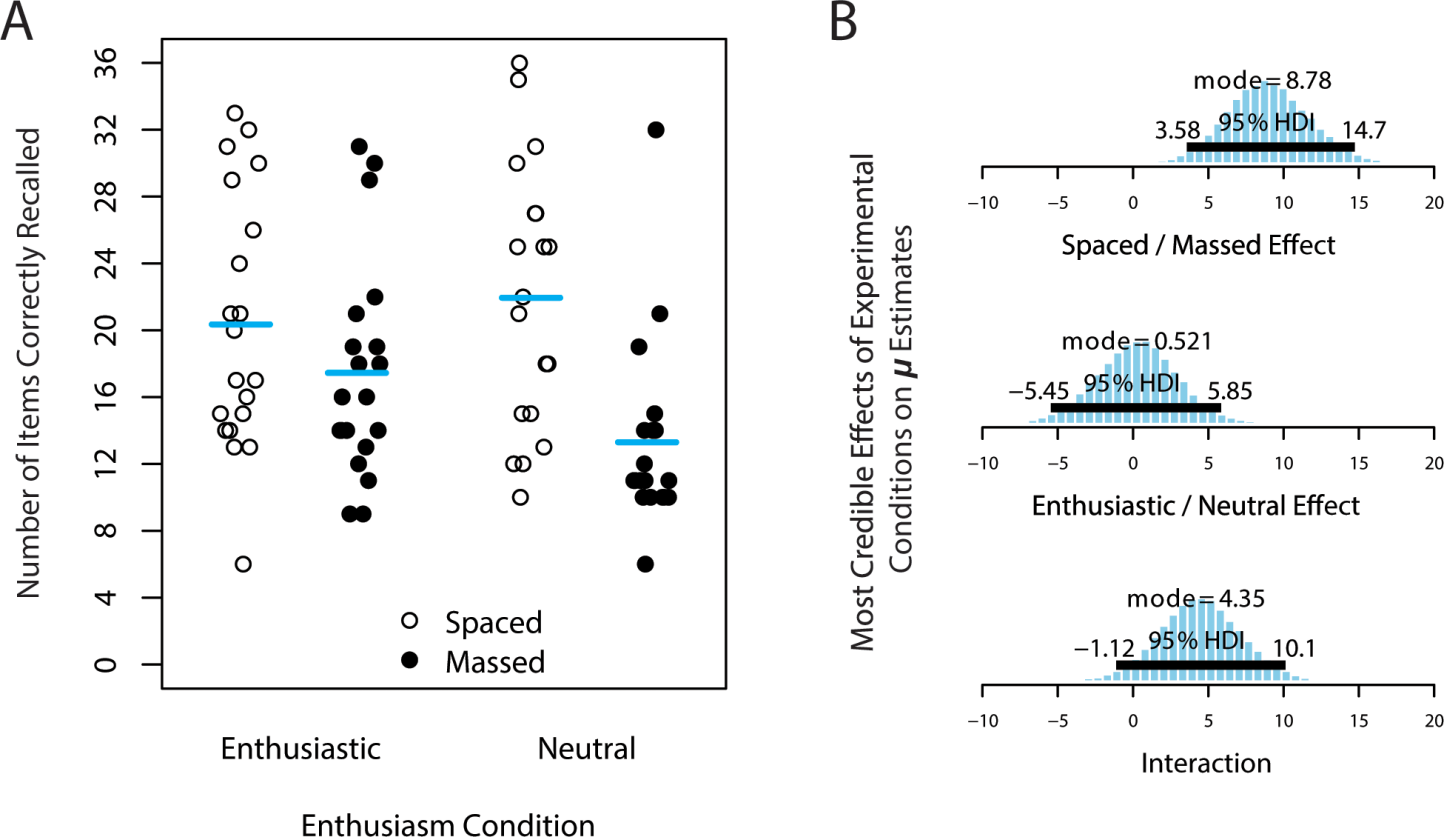
Number of items correctly recalled in Experiment 1. (A) Number of items correctly recalled (out of 36 possible) in Experiment 1. Each participant’s raw score is a point; horizontal bars mark the arithmetic mean of correct recall in each condition. (B) Posterior estimates of experimental effects on *μ*, the central tendency of item recall, for (top) spaced—massed, (middle) enthusiastic—neutral, and (bottom) the interaction between these two factors (benefit of enthusiasm in massed—benefit of enthusiasm in spaced). The 95% highest density interval (HDI) shows the range of values that contains 95% of the posterior distribution, so that the values inside the HDI are more probable than those outside.

On average, out of 36 pairs, participants provided the correct English translation for 18.2 items (50.6%; *SD* = 7.6), provided incorrect responses on 13.5 items (37.5%; *SD* = 7.7), and left blank the remaining 4.3 items (11.9%; *SD* = 6.3). Fig 2A illustrates the number of correctly recalled items for each of the experimental conditions.

Our analysis model included group-level parameter estimates of average items correctly recalled, *μ*, for each of the four combination of conditions (enthusiastic/massed; enthusiastic/spaced; neutral/massed; and neutral/spaced). Collapsing across sequence conditions (spaced and massed), the difference between enthusiastic and neutral *μ* estimates at every step in the MCMC chains creates a posterior distribution of memory improvement due to enthusiasm. The mode of this distribution is 0.521 items (95% HDI: -5.45 to 5.85 items), suggesting that the most credible estimates of the main effect of enthusiastic instructions are close to zero. Similarly, collapsing across enthusiasm conditions, we can calculate the difference between spaced and massed parameter estimates, which yields a posterior distribution with a mode of 8.78 items (95% HDI: 3.58 to 14.7 items). From this posterior distribution, we can infer that there was a 3 to 15 item improvement when the study sequence was spaced compared with massed. Contrasting these main effects, the effect of sequencing is greater than the effect of enthusiasm (difference mode = 8.01 items; 95% HDI: 0.697 to 18.1 items). And lastly, by taking the difference of differences between these four parameters (benefit of enthusiasm in massed—benefit of enthusiasm in spaced), we can also estimate the posterior distribution of the interaction effect between these two factors, which has a mode of 4.35 items (bigger effect of enthusiasm in the massed condition than the spaced condition; 95% HDI: -1.12 to 10.1 items), but given that the 95% HDI spans zero, it is unclear whether the interaction effect is reliably different from zero, given these data. The full posterior distributions of these differences in parameter estimates are shown in Fig 2B.

**Fig 3:**
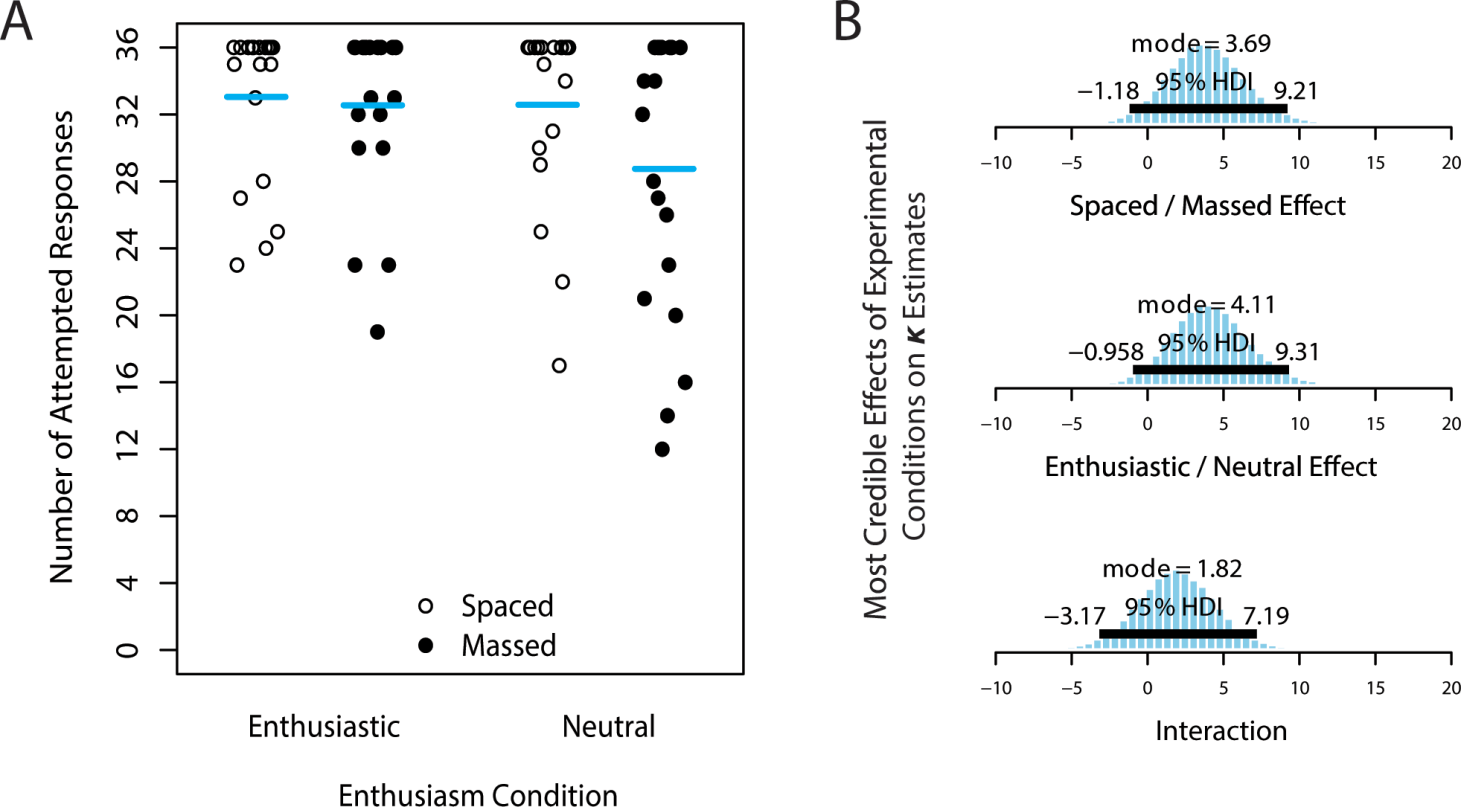
Number of attempted responses in Experiment 1. (A) Number of attempted (non-blank) responses (out of 36 opportunities), irrespective of response correctness. Each participant’s number of attempted responses is a point; horizontal bars mark the arithmetic mean of the dataset. The modal value in all conditions is 36 (note the high concentration of points at the top of the chart). (B) Posterior estimates of experimental effects on *κ*, a concentration parameter that is inversely proportional to the “spread” of attempted responses (*κ* values increase as the data are more concentrated near the mode of 36). Contrasts are shown for (top) spaced—massed, (middle) enthusiastic—neutral, and (bottom) the interaction between these two factors (increased concentration of enthusiasm in massed—increased concentration of enthusiasm in spaced).

The number of correctly-recalled items is an objective measure of learning, but alternative recall measures might also be sensitive to the enthusiasm manipulation. Specifically, we also analyzed total attempted responses, the number of non-blank answers provided during recall (combining both correct and incorrect responses). The majority of participants (51.9%) provided some response to all of the 36 Indonesian word prompts, but there was some variability in the “spread” of the number of attempted responses across conditions (illustrated in Fig 3A). In our analysis model (which can similarly be applied to the total number of attempts), the variance of a distribution is estimated by *κ*, the concentration parameter of the beta distribution, a measure of how “bunched” the observations are around the mode [59]. Given that the mode of the distribution is at the maximum value, an increase in *κ* indicates an increase in the number of attempted words, and naturally accounts for the ceiling effect in the current data. In other words, considering that the mode in all four experimental conditions (parametrized by *μ*) is 36, only the concentration parameter (*κ*) would be expected to account for differences between conditions in the total number of responses (the arithmetic mean of the dataset is not estimated in this analysis model; only mode and concentration). Similar to the previous analysis of *μ* for correctly-recalled items, we calculated differences in *κ* estimates between levels of each factor, creating posterior distributions of the factor’s effect on total attempted responses. These posterior distributions are shown in Fig 3B. The most credible estimate of the spacing effect on *κ* is 3.69 (95% HDI: -1.18 to 9.21), and for the effect of enthusiasm, *κ* is estimated at 4.11 (95% HDI: -.958 to 9.31). Because both posterior distributions overlap zero, neither of these trends reached our decision threshold for being reliably different from no effect, although they do raise interesting questions for further examination.

### Discussion

Enthusiastic task instructions did not have any main effect on memory performance in Experiment 1. This absence of an effect of enthusiasm lies in stark contrast to the robust observed effect of study sequence (spaced vs. massed repetitions of an item) on recall, which has been well-established in the literature (for a review, see [38]). One obvious inference then, which cannot be rejected, is that enthusiastic instruction does not improve learning. However, there are other possible interpretations.

Perhaps the experimental manipulation of enthusiasm was not sufficiently strong to spur a detectable improvement. After all, participants’ subjective ratings of Ashley’s enthusiasm and her memorability were just slightly higher in the enthusiasm condition, insufficient to determine a nonzero difference estimate. In real classrooms, repeated and extended exposure to an enthusiastic instructor may yield a more robust effect. Within the current study design, using short video clips of a simulated instructor, should we even be able to observe such an effect of instructional enthusiasm? We believe so. Using short videos to manipulate and control instructor enthusiasm is typical in this literature (e.g., [16]), short instructional videos are common and useful tools in contemporary blended learning contexts [66], and in the current study, considering that the video was the only source of task instructions, performance strongly hinged on participants’ attention to the videos.

Another possibility is that enthusiasm interacts with task difficulty. We observed a marginal interaction such that the benefit of enthusiasm was greater in the massed condition than the spaced condition. Considering that spaced practice implicitly requires greater study effort [67–69], there might be less opportunity for enthusiastic instructions to benefit learning; but for massed practice, there might be room for enthusiasm to impel students to try harder. However, the posterior distribution for this interaction effect overlapped zero and values close to zero, so this inference fails to receive clear support from the present data.

One possibility then, is that the enthusiasm manipulation had some small effect on performance, perhaps isolated to the more difficult massed condition, but the sample was not large enough to reliably detect it. Similarly, the results hinted toward a small effect of enthusiasm on total attempted responses (perhaps enthusiasm causes students to guess more often), but the most credible parameter estimates included zero and values close to zero. Conventional (frequentist) measures of power suggest that, in order to achieve an 80% likelihood of observing a significant interaction between enthusiasm and study sequence, we would need approximately twice as many participants. This is based on the assumption that the effect size of the interaction is close to what we observed in this study.

The monetary bonus that participants received for correct recall represents another possible culprit for the lack of effect of enthusiasm. Enthusiasm was predicted to improve motivation [34], but it is possible that current participants were principally motivated by the financial incentive. Indeed, past researchers have suggested that an instructor’s expressiveness will only improve learning when students have no other extrinsic incentive to achieve [24, 25], and participants’ self-assessments of their own level of motivation were practically equivalent between enthusiasm conditions. We opted to use financial incentives in Experiment 1 to increase the likelihood that student participants would study hard during the first session and return to the lab for the second session, but these may have reduced the potency of enthusiasm’s effect on task motivation.

In summary, there were several possible explanations for the failure to observe an effect of enthusiasm on memory performance: financial incentives outweighing the motivating effect of instructor enthusiasm, insufficient sample size to reliably detect effects, and small differences in participants’ subjective ratings of the enthusiasm manipulation. Experiment 2 was designed to address these issues, testing the possibility that an effect of enthusiasm might become apparent with a larger sample size, and without extrinsic incentives (which might result in a more robust manipulation of enthusiasm). Moreover, considering that the enthusiastic instructions, in total, were 31 seconds (12.6%) longer than the neutral condition, we also aimed to equate the lengths of these videos, eliminating the possibility that length of instruction would affect task performance.

## Experiment 2 (Confirmatory study)

Experiment 2 was similar to Experiment 1, but with three important differences: (1) We increased the sample size, more than doubling the number of participants; (2) We removed the monetary incentive for memory performance; and (3) We produced new videos that better-equated the lengths of enthusiastic and neutral instructions, and to generalize Experiment 1’s results to a new instructor. We preregistered the study on OSF, with the plan to use the same analysis model used for the proportion of correct responses in Experiment 1 with a larger sample size (recorded at https://osf.io/qmg95/). All materials and data for Experiment 2 are available at http://osf.io/j2h8y/.

### Method

#### Ethics statement

The experimental procedures, materials, and recruitment protocols were approved by the Indiana University Institutional Review Board. All participants provided informed consent electronically at the start of the experiment.

#### Participants

Two hundred and forty-one undergraduate students at Indiana University volunteered in exchange for credit toward their Introductory Psychology experiment participation requirement. We excluded 42 participants because they did not return for the second session (N = 14), the unique subject identifier was accidentally assigned to more than one participant (N = 3), the dataset was incomplete due to skipped trials or questions (N = 14), they spoke Indonesian (N = 3), or they were enrolled in Introductory Psychology with Benjamin Motz (N = 8; one of the authors of this study, and the simulated instructor for Experiment 2; see *Video Recorded Instructions*, below). After exclusions there were 199 participants, 111 females (by self-report; 55.8%), with an average age of 18.9 years (*SD* = 1.2). One hundred sixty nine of these participants (85%) reported being native English speakers, and the remaining non-native English speaking participants had been speaking English for an average of 11.1 years (*SD* = 4.8).

#### Stimuli

We used the same English-Indonesian word pairs as Experiment 1, but to improve the sensitivity of this stimulus set, word pairs that were successfully recalled by at least 75% of participants in Experiment 1 were replaced with new pairs from [54]. There were 9 pairs from Experiment 1 that met this criterion, each close cognates (e.g., Photocopy / Fotokopi). We selected 9 replacement pairs that avoided any such surface similarity between the English and Indonesian tokens.

#### Video recorded instructions

We edited the scripts used in Experiment 1 so that the lengths of the enthusiastic and neutral scripts were evenly matched, and to remove reference to any financial bonuses for correct recall. Instead of extrinsic incentives, both scripts simply asked participants to invest effort in the task because it would benefit science, and because the researchers would appreciate it.

With these new scripts came the opportunity to recreate the videos with a new instructor, seeking further generalizability. Rather than recruiting another actor, one of the authors of this article (Motz), an experienced college teacher, memorized the scripts and recorded himself delivering the instructions, enthusiastic and neutral, in the same manner as described in Experiment 1. These videos were recorded in a small classroom in the Psychology Building (see Fig 1 for representative stills; the individuals shown in this figure have given written consent to publish these images).

The total duration of all recorded video segments in the enthusiastic condition was 5 minutes 31 seconds, and 5 minutes 15 seconds in the neutral condition. This small difference in duration is caused by expressive pauses in the enthusiastic videos, not because the enthusiastic videos had more content or information.

#### Procedure

The procedure for Experiment 2 was identical to Experiment 1, except that participants did not receive cash bonuses for performance, and the simulated instructor’s name was updated in questionnaire items.

#### Bayesian data analysis

The analysis models and method for Experiment 2 were identical to Experiment 1. To provide the most conservative examination of experimental effects, we used the same vague priors as Experiment 1.

### Results

The enthusiasm manipulation had a stronger effect on participants’ ratings in Experiment 2 than in Experiment 1. Participants in the enthusiasm condition clearly rated the simulated instructor, Ben, as being more enthusiastic (*M* = 3.5, *SD* = 0.65; neutral, *M* = 2.8, *SD* = 0.86; difference mode = 0.819; 95% HDI: 0.627 to 1.01) and more memorable (*M* = 3.2, *SD* = 0.93; neutral, *M* = 2.5, *SD* = 0.95; difference mode = 0.717; 95% HDI: 0.448 to 0.983). There was also a directional trend for participants to report feeling slightly more motivated to perform well in the enthusiastic condition (*M* = 3.0, *SD* = 0.80) than the neutral condition (*M* = 2.8, *SD* = 0.69), perhaps due to the removal of the cash bonuses for performance, although credible estimates of this group difference overlapped zero (mode = 0.164; 95% HDI: -0.0408 to 0.372).

**Fig 4:**
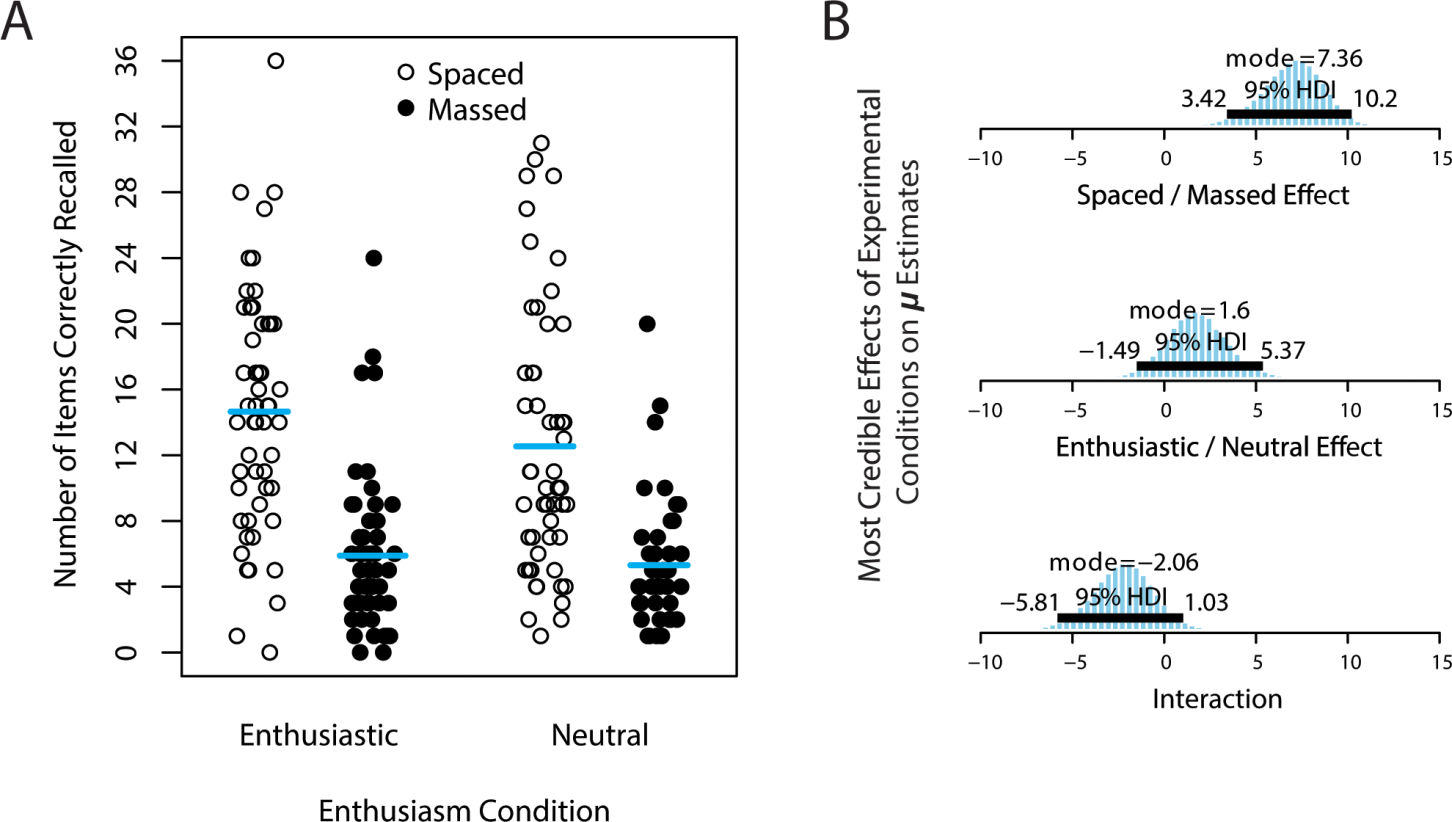
Number of items correctly recalled in Experiment 2. (A) Number of items correctly recalled (out of 36 possible) in Experiment 2. Each participant’s score is a point; horizontal bars mark the arithmetic mean of correct recall in each condition. (B) Posterior estimates of experimental effects on *μ*, the central tendency of item recall, for (top) spaced—massed, (middle) enthusiastic—neutral, and (bottom) the interaction between these two factors (benefit of enthusiasm in massed—benefit of enthusiasm in spaced). The 95% highest density interval (HDI) shows the range of values that contains 95% of the posterior distribution, so that the values inside the HDI are more probable than those outside.

Also likely stemming from the lack of financial incentives, as well as the removal of the most frequently-recalled items, recall accuracy was lower in Experiment 2 than Experiment 1. Across all conditions, participants correctly recalled only 9.8 items (27.2%; *SD* = 7.6), provided incorrect responses on 20.7 items (57.6%; *SD* = 10.1), and left the remaining 5.5 items empty (15.2%; *SD* = 8.3). Fig 4A illustrates recall accuracy for the four different conditions.

In spite of the increased sample size (with accordingly narrower HDIs), and participants’ stronger subjective ratings of enthusiasm, the most credible estimates of the effect of enthusiasm on recall accuracy still overlapped zero (mode = 1.6; 95% HDI: -1.49 to 5.37). Although this posterior distribution is shifted slightly in the direction of a very small positive benefit of instructional enthusiasm, this benefit was not reliably different from zero effect. In contrast, study sequence again had a robust main effect, our best estimate is that participants who were assigned a spaced sequence correctly recalled 7.36 more items than those who were assigned a massed sequence (95% HDI: 3.42 to 10.2). Although we observe a large and reliable effect of study sequence, the slight positive shift in the effect of enthusiasm meant that the difference between these main effects (sequence minus enthusiasm) overlapped zero (difference mode = 5.51 items; 95% HDI: -0.305 to 10.0). Also, the posterior distribution of the interaction between sequence and enthusiasm again spanned zero (mode = -2.06; 95% HDI: -5.81 to 1.03), and if anything, the most credible estimates suggest a reversal from Experiment 1 (improvement due to enthusiasm is now smaller in the massed condition than the spaced condition).

**Fig 5:**
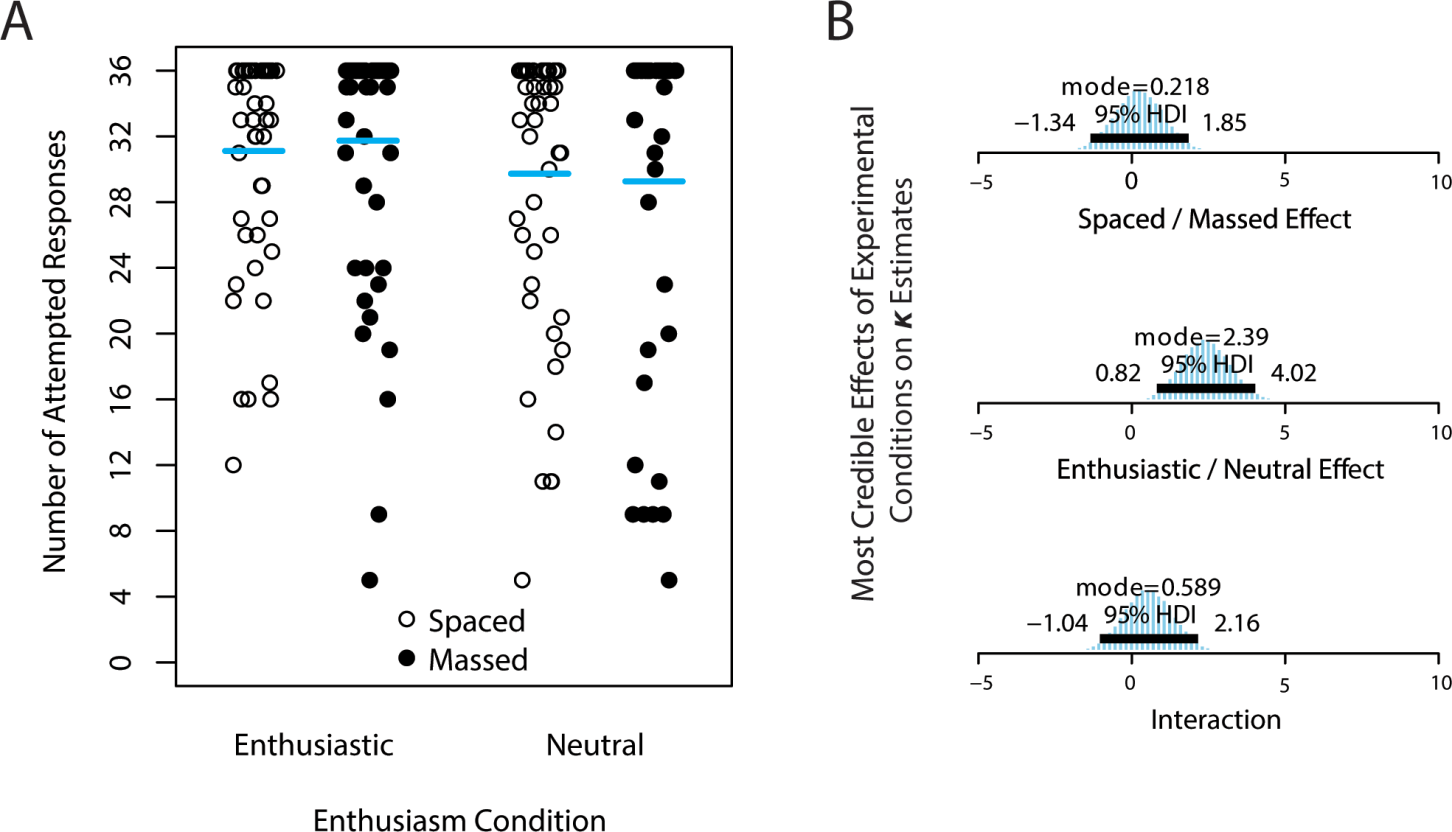
Number of attempted responses in Experiment 2. (A) Number of attempted (non-blank) responses (out of a maximum 36), irrespective of response correctness, in Experiment 2. Each participant’s number of attempted responses is a point; horizontal bars mark the arithmetic mean of the dataset. The modal value in all conditions is 36 (note the high concentration of points at the top of the chart). (B) Posterior estimates of experimental effects on *κ*, a concentration parameter that is inversely proportionate to the “spread” of attempted responses (*κ* values increase as the data are more concentrated near the mode of 36). Contrasts are shown for (top) spaced—massed, (middle) enthusiastic—neutral, and (bottom) the interaction between these two factors (increased concentration of enthusiasm in massed—increased concentration of enthusiasm in spaced).

Although participants had lower overall accuracy in Experiment 2, the majority (50.3%) still provided a response to all 36 of the Indonesian word prompts. The remaining 49.7% of participants left at least one item blank. Fig 5A illustrates the number of attempted responses across all conditions. We conducted an exploratory analysis on the likelihood of a participant providing an answer, regardless of accuracy. This is measured by *κ*, the concentration of attempts near the maximum of 36 items. Posterior estimates of *κ* suggest that enthusiasm increased the likelihood of providing a response (mode of *κ* differences = 2.39; 95% HDI: 0.82 to 4.02), but *κ* was not reliably affected by study sequence (mode = 0.218; 95% HDI: -1.34 to 1.85), nor was there an interaction between study sequence and enthusiasm (mode = 0.589; 95% HDI: -1.04 to 2.16).

### Discussion

Experiment 2 was a confirmatory study, preregistered to examine the hypothesis that an effect of instructional enthusiasm on memory performance, or an interaction between enthusiasm and study sequence, would become apparent with a larger sample. These hypotheses were not supported.

We failed to observe any reliable effect of enthusiastic instructions on learning performance despite a large sample size, and also despite manipulation checks confirming that the intervention had a strong effect on participants’ subjective ratings of the instructor’s enthusiasm and the memorability of the instructions. And enthusiasm was not psychologically inert. Although there was no effect on recall accuracy, our exploratory analysis found that enthusiastic task instructions caused participants to provide more answers to the Indonesian word prompts (or, in other words, they were less likely to leave items blank), as we observed the distribution of items answered to be more concentrated around the maximum value of 36 in the enthusiasm conditions. We observed this same directional pattern in Experiment 1 (although the 95% HDI overlapped zero), but in Experiment 2, with increased power, enthusiastic instructions reliably increased the likelihood of providing a response. There was no penalty for incorrect responses in the current study, and since there was no difference in the number of correctly-recalled items, the additional responses in the enthusiasm condition were incorrect (many were either incorrect cognates, such as ‘liburan’ translated as ‘librarian’ instead of ‘holiday,’ or words that would be correct translations on other items).

As an alternative to merely leaving an item blank, an increased tendency to provide a response, even if incorrect, is evidence of a more liberal response criterion [70]. In classic memory research, experimental manipulations of task motivation have been shown to affect this response criterion, resulting in increased response attempts even in the absence of improvements in recall accuracy. Explicit instructions and encouragement to provide more responses [53, 71, 72] and financial incentives [52] may increase the number of responses, but past studies have shown these are not typically accompanied by increased quality of the memory trace or accuracy of the responses [70, 73]. It would seem that enthusiastic instruction has an effect that is analogous to encouraging increased responses, incentivizing participants to be more engaged with the task, less likely to leave items blank, but not providing any measurable advantage for learning or memory.

## General discussion

Based on the results of the current study, we conclude that if there is any isolatable, direct effect of instructional enthusiasm on recall accuracy, it is small and unreliable. Across two carefully controlled experiments, we found that participants exposed to an enthusiastic instructor do not demonstrate reliable improvements in how much they learn during a memory task.

Since Rosenshine’s [31] early claims of “strikingly consistent results” (p. 506) relating enthusiasm to pupil achievement, there have been two divergent trends in the literature. On the one hand, experimental research has severely disrupted this consistency—null effects of enthusiastic instructions on test performance, such as those reported in the current study, have steadily mounted [20–24, 27–29, 74]. On the other hand, students and educators have both become more steadfast in their endorsement of enthusiasm as a crucial aspect of effective teaching [12].

How can we reconcile enthusiasm’s broad popularity in education with our failure to observe a consistent benefit of an instructor’s enthusiasm on learning? Perhaps the answer lies in accepting that motivated task behavior does not necessarily lead to memory improvement.

### A dissociation between learning and motivation

Stated broadly, the current results suggest that enthusiasm, of the kind manipulated here, has little practical value for memory performance. If the goal of an academic exercise is to help a student remember the to-be-learned material, enthusiastic instructions may not have direct benefit toward this goal. Our results suggest that learners would probably reap better outcomes with an optimized studying sequence (or perhaps other cognitive strategies [47]) than by exposure to an expressive and dynamic instructor. Lest these conclusions should seem surprising to teaching enthusiasts, recent research has gone even further to demonstrate that an instructor’s fluency (the ease with which students perceive to-be-learned material) has no direct effect on learning [75, 76].

Even though instructor enthusiasm has no effect on recall accuracy, results from Experiment 2 indicate that it still had motivational effects on task behavior, increasing self-reported interest in the instructor’s presentation, and increasing the number of responses provided during test. Our results support and extend those of Bettencourt et al.[21], who also observed evidence of increased student motivation toward task behavior, but no effect on test performance, due to an otherwise robust experimental manipulation of instructor enthusiasm. Patrick et al. [34] similarly found that enthusiastic instructions had a contagious effect on students’ self-reported levels of motivation during a learning activity, but there was no effect on a behavioral measure of self-directed learning. Rather than accept a dissociation between motivation and achievement, these past researchers attributed their mixed results to methodological nuance. The folk theory that enthusiastic teaching begets student motivation, increasing interest and attention, which then improves learning [31], was too compelling to dispute [21, 51]. However research in cognitive psychology has repeatedly shown that learning performance does not necessarily benefit from increased interest [77, 78] or increased motivation [52, 79].

The current results add to mounting evidence indicating that a motivated student is not necessarily a *learned* student—a fact that may intuitively resonate with teachers who have encountered students who try hard, but seem unable to accurately recall the material. Motivation alone does not improve test performance. Rather, as observed in the current study, motivation may instead manifest as increased self-reported interest in an activity, and as being more likely to respond to explicit prompts. These are nontrivial outcomes, but recall accuracy does not necessarily hinge on them.

### Implications

The present lack of an influence of instructor enthusiasm on memory accuracy may, of course, be restricted to either our manipulation of enthusiasm, or to the learning task. Prolonged, chronic instructor enthusiasm may have an influence on performance, may affect a more meaningful learning task, or may have isolated effects on students depending on their motivations and goals. Nonetheless, short “doses” of instructor enthusiasm (such as those commonly manipulated in this line of research) are likely to be of increasing practical relevance in contemporary educational environments where the instructor is not directly supplying learning materials, but rather is directing students’ active learning efforts (discussed in [34]). It is noteworthy, then, that we observed divergent effects when concurrently manipulating instructional behaviors and learning materials; the instructor’s enthusiasm did have positive influences on learners’ test effort and self-reports of engagement, but only the properties of the learning materials (item sequence) reliably affected test performance.

Enthusiasm’s relationship to student achievement (commonly operationalized as test performance) is not supported. However, enthusiasm is not without virtue (unless the reader believes that the ultimate goal of education is merely to improve test performance). Its effect on student motivation may serve important practical purposes in the classroom, such as seeding interest in a topic, or engaging the learner in an activity. Just because it does not significantly improve recall of information does not imply that educators should jettison enthusiasm from schools, from teaching evaluation instruments, or from the advice given to new instructors. Claims regarding enthusiastic teaching’s benefits should, however, be limited to improvements in task motivation and enjoyment (which are well-supported by empirical research, including the current study); and such claims should not extend to improvements in test performance. Education is a fundamentally social activity, and teachers’ behaviors have important consequences beyond memorization [80–83]. This complexity is inherent in educational systems, and applications of memory research will benefit from future experimental examinations of the relationship between instructional techniques and student learning [84].
